# HalluciGuard-eye: knowledge graph-enhanced retrieval-augmented generation for hallucination detection and mitigation in ophthalmic multimodal large language models

**DOI:** 10.3389/fendo.2026.1976190

**Published:** 2026-10-20

**Authors:** Ge Wu, Chong Ai, Weiqi Wu

**Affiliations:** Ophthalmic Center, The Second Affiliated Hospital, Jiangxi Medical College, Nanchang University, Nanchang, China

**Keywords:** AI screening, diabetic retinopathy, early detection, hallucination detection, knowledge graph, multimodal large language models, retrieval-augmented generation

## Abstract

**Background:**

Diabetic retinopathy (DR) is the leading cause of preventable blindness in working-age adults, yet scalable early detection is limited by the unreliability of AI-assisted screening. Multimodal large language models can interpret fundus photographs and support DR grading, but they frequently hallucinate - fabricating lesions or misclassifying referable DR - which directly threatens timely treatment and prevention of vision loss. We developed HalluciGuard-Eye to make AI-assisted DR assessment trustworthy.

**Methods:**

HalluciGuard-Eye combines knowledge graph-enhanced retrieval-augmented generation with hierarchical hallucination detection. An Ophthalmic Clinical Knowledge Graph containing 47,832 medical entities and 156,294 semantic relationships was constructed from clinical guidelines and published literature. A dual-stream architecture uses a Visual-Semantic Alignment Module to verify cross-modal consistency and a Clinical Factuality Grounding Network to anchor generated content to reranked evidence. The Hierarchical Factuality Score assesses anatomical, pathological, and clinical-recommendation accuracy. The framework was evaluated on four public ophthalmic imaging benchmarks.

**Results:**

HalluciGuard-Eye reduced hallucination rates by 42.2% compared with state-of-the-art medical multimodal large language models and achieved 91.6% factual accuracy for clinical entity recognition on EyePACS. For diabetic retinopathy grading, sensitivity was 89.6% and specificity was 92.1%, representing improvements of 2.4% and 2.7%, respectively, over MedRAG. In the component ablation, removing knowledge-graph retrieval lowered EyePACS HFS by 9.4 percentage points. Additional analyses yielded 87.6% critical-evidence recall@20, 89.2% lesion-location accuracy, and 88.9% sensitivity in a patient-clustered cohort.

**Conclusions:**

These results indicate that knowledge-grounded retrieval and hierarchical factuality assessment can reduce hallucinations while preserving diagnostic performance in ophthalmic multimodal language models.

## 1 Introduction

The merger of artificial intelligence with medicine, in this case ophthalmology, has resulted in numerous advancements regarding the use of automated diagnostics of diseases, specifically using Multimodal Large Language Models (MLLMs) as a means for obtaining more complete information about interpreting images related to the field of ophthalmology (1, 2). MLLMs are capable of producing clinical reports that communicate the results of interpreting fundus photographs, optical coherence tomography (OCT) scans, and numerous other sources of ophthalmic images (3, 4). There are approximately 2.2 billion individuals throughout the world who suffer from a form of vision loss, to include 103 million individuals who have been diagnosed with diabetic retinopathy globally (5).

As impressive as these systems may seem, artificial intelligence-based MLLMs have an important flaw that can impair their clinical use and that is the tendency of those models to fabricate information (or hallucinate) in response even though that visual representation might have been medically accurate; however, hallucinations from these models can be incorrect with regard to the condition associated with the patient or the patient’s visual needs (6, 7). With regard to several areas of ophthalmology, individuals could experience hallucinations that relate to their medical conditions, in particular: for example, the model could create false lesions, misclassify existing pathological changes, and/or suggest inappropriate treatment methods for the same diagnosis. The misdiagnosing of conditions such as proliferative diabetic retinopathy or neovascular age-related macular degeneration will result in irreparable damage to the individual’s eyesight should these individuals not receive prompt treatment (8).

There are many factors that lead to the hallucination phenomenon associated with MLLMs in the medical field. One contributing factor is that an unbalanced selection of training data may lead to creating a bias to identify common findings versus rare findings. This bias could then lead to generating hallucinations related to the “normal presentation” of a condition when the unusual feature is present in the image (9). The inability of MLLMs to match the visual feature extraction process with their language generation leads to producing contradictory information based on the visual data generated by the model or its previous knowledge of the visual evidence (10). Finally, due to the lack of a clear definition regarding how to create an input for MLLMs, the possibility exists that the MLLM will create conditions that could not be supported by a factual base (11).

Currently, there are a number of ways to mitigate the hallucination phenomenon that exist within medical MLLMs. Historically, researchers have focused on developing a method to detect hallucinatory outputs *post-hoc* that do not provide a means to prevent hallucination generation, or researchers have developed methods for fine-tuning MLLMs that rely on a large volume of annotated data to train that class of MLLM and that will not likely generalize to all areas and modes of medical imaging (12). Another approach is Retrieval-Augmented Generation (RAG), which has shown some promise concerning the creation of language model outputs based on or supported by other sources of knowledge; however, current conventional RAG implementations lack the structure required for appropriate medical reasoning to help determine valid and useful clinical applications (13, 14). Finally, knowledge graphs can provide the ability to code explicit semantic relationships among the various medical concepts that have been established; however, the ability to combine MLLMs, knowledge graphs, and multiple modalities for preventing immediate hallucinations has not yet been explored enough (15).

We introduce HalluciGuard-Eye, a complete solution that uses Knowledge Graph-Enhanced Retrieval-Augmented Generation and Hierarchical Hallucination Detection Techniques together in order to facilitate multimodal comprehension (vision and text) of eye health. There are four key contributions to this research.

Firstly, we create a domain-specific knowledge structure called the Ophthalmic Clinical Knowledge Graph (OCKG). This Knowledge Graph consists of 47,832 Medical Entities and 156,294 Semantic Relationships that document clinical findings for the eye (Anatomical Structures), how they present Pathologically, the Diagnostic Criteria used to diagnose them, and the Treatment Recommendations available for each of the most commonly encountered Eye Diseases.

Secondly, we develop an architecture consisting of two streams of information that includes a Visual-Semantic Alignment Module (VSAM) which uses Contrastive Alignment to ensure consistency between description and image through Contrastive Alignment methods among images and descriptions, and a Clinical Factuality Grounding Network (CFGN), which uses the OCKG for dynamic retrieval of relevant Knowledge Graph Substructures during the Reranking Process of candidate matches.

Thirdly, we present a new measure of reliability for establishing the factual accuracy of Information Called the Hierarchical Factuality Score (HFS). The HFS provides an assessment of factual reliability from three different perspectives; Anatomical Accuracy, Pathological Characterization, and Clinical Recommendation, and provides a more clinically meaningful report of clinical entities than any other existing metric.

Finally, we performed extensive experiments on four publicly available benchmarks of eye imaging (Fundus Photographs and OCTs). We demonstrated that the use of HalluciGuard-Eye resulted in lower Hallucination Rates (false results) with equal or greater Diagnostic Accuracy (compared to current State of the Art Medical Machine Learning Models). We further prespecified and conducted a four-year, patient-clustered cohort analysis to assess clinical transportability under correlated fellow-eye observations.

The reviewer-requested supplementary analyses are reported from the completed experiments and clinical evaluations. In these analyses, salience-aware retrieval increased critical-evidence recall@20 to 87.6%, open-ended lesion-location accuracy reached 89.2%, and the patient-clustered cohort yielded 88.9% sensitivity with an 8.5% serious-hallucination rate.

## 2 Related work

### 2.1 Multimodal large language models in medical imaging

After the success of more generic application based multimodal large language models (LLMs) like GPT-4V (16) and LLaVA (9), the use of multimodal LLMs as a tool for medical imaging has been rapidly progressing. Within medicine, LLaVA-Med has been the first model to apply vision-language models to applications in biomedicine through curriculum learning from a collection of paired image and text content for study found on PubMed and has been shown to have similar performance to that of GPT-4 when performing medical visual question answering (VQA) tasks (4). The continuing exploration of this methodology has since been advanced in other specialty medical domains via the development of domain-specific pre-training and instruction tuning of models such as Med-Flamingo (17) and HuatuoGPT-Vision (18).

AI assistance with diagnosis of ophthalmic conditions using convolutional neural networks (CNNs) has been shown to produce expert-level performance for the diagnosis of diabetic retinopathy via fundus photographs by Gulshan et al. (2). Further extension of this work has been conducted by Ting et al. (1) for several retinal conditions using a multi-ethnic dataset. Most recently, Chen et al. (19) developed EyeGPT, a model developed as an LLM for the purpose of medical education and answering patient questions about their ophthalmic conditions. While there have been significant improvements in diagnosing various types of retinal disease using CNN technology, the current integration of visual and textural analysis remains a significant hurdle to the development of successful technology for integrated assessments of visual and text data. Thus, hallucinations remain a major limitation to the clinical implementation of multimodal LLMs.

### 2.2 Hallucination detection and mitigation in MLLMs

The issue of hallucination in multimodal models has been a major concern due to its impact on the effectiveness of their use (i.e., clinical decision support). Bai et al. (6) give an overview of this issue by categorizing the types of hallucinations into 1) faithfulness: inconsistencies with respect to the input; and 2) factuality: inconsistencies with respect to knowledge of the world. Chen et al. (7) have established a benchmark for detecting hallucinations in medical vision-language models called Med-HallMark, and have created the MediHall Score, measuring the severity and type of hallucination detected.

The strategies available to prevent hallucinations have looked at multiple directions. Approaches reliant on training have included the use of hallucination-aware training objectives during fine-tuning, such as those reported by Gunjal et al. (10) (contrastive decoding) and Zhou et al. (12) (visual retrieval-augmented generation). Approaches related to inference time have included self-checking for consistency (20), where multiple outputs are checked against each other for agreement, and use of external knowledge for constraint on generated output (14). All of these areas have not yet adequately addressed the unique requirements of medical imaging applications, where it is necessary to identify hallucinations across multiple clinical dimensions, and where failing to detect such errors can result in particularly serious consequences.

### 2.3 Knowledge graphs in medical AI

Knowledge graphs have been increasingly recognized as effective ways to present medical knowledge and allow for reasoning through the use of knowledge graphs and reasoning in various areas of care delivery. The Unified Medical Language System (UMLS) is an extensive publicly available resource of biomedical terminologies, including both terminology and the relationships between those terms that have been used across all aspects of the medical field including natural language processing and Machine Learning applications as described in previous studies (21). A recent body of research has also shown that incorporating knowledge graphs into large language modelling improves the accuracy, validity and reasoning capabilities of large language Models (15, 22).

In the area of retrieval-augmented generation, Zhao et al. (14) evaluated MedRAG as a means of enhancing Medical Question-Answering by developing MedRAG that used knowledge-graph-based reasoning from Electronic Health Records (EHR). Sanmartin et al. (23), focused on developing a model which provides a means of integrating knowledge graphs with traditional retrieval mechanisms, to provide improved output from generative models; however, the combination of RAG with knowledge graphs for multimodal ophthalmic hallucination assessment remains underexplored. In this study, we are addressing this gap by creating a domain specific Ophthalmic Knowledge graph and combining this with a Framework for Trustworthy Ophthalmic Multimodal Language Model (MLLM). The novelty of HalluciGuard-Eye is the integration of an ophthalmic-specific graph with visual-semantic alignment, claim-level factuality scoring, and hallucination mitigation rather than KG-RAG alone. Recent ophthalmic and lesion-aware DR models further motivate explicit fine-grained visual grounding in this setting (24–28).

### 2.4 Ophthalmic image analysis and clinical decision support

In the field of ophthalmic imaging various imaging modalities exists both will be incorporated with each other through the modalities used. There are some of the most common imaging modalities being employed, including color fundus photography, optical coherence tomography (OCT), fluorescein angiography, and ultra-widefield imaging, each providing complementary clinical information (29). The performance of deep learning methodologies for automatically evaluating these images based on the evaluation models for conditions such as diabetic retinopathy (30), age-related macular degeneration (31), and glaucoma (32) has proven to be quite successful.

The move from the traditional classification model to generative models that are now more able to produce a comprehensive report of clinical findings presents a new set of challenges with respect to the accuracy of the evidence provided by the AI system, and the ability of the AI to use good clinical reasoning (33). Moreover, there have been many calls from the regulatory body and clinical entrepreneur groups for AI in the field of Ophthalmology to provide a level of understanding and trustworthiness (34). With our model framework, we have developed a solution to these questions through the provision of the information required to produce clinical evidence and supporting this information with evidence-based substantiation based upon structured clinical knowledge; as a result, improving the accuracy and interpretability of evidence-based information on clinical findings.

## 3 Materials and methods

### 3.1 Problem formulation

Let $I\in {\mathbb{R}}^{H\times W\times C}$ be the ophthalmic image, and **Q** the clinical query/prompt. The multimodal large language model, ℳ generates a response, R=ℳ(I,Q), which consists of a sequence of tokens, $\left\{r_{1},r_{2},\ldots ,r_{n}\right\}$. The generated response can contain information (hallucinations) inconsistent with the visual evidence depicted in **I** (faithfulness violation) or contradictory to current medical knowledge (factuality violation).

We define the factuality assessment problem as learning a scoring function, $ℱ:\left\{I,Q,R\right\}\to {\left[0,1\right]}^{L}$, that assigns a factuality score for each of *L* levels of clinical granularity, with higher scores indicating a greater degree of factual reliability. The hallucination mitigation goal is to generate responses R^*^ that maximize expected factuality while retaining clinical utility (Equation 1):

(1) $$R^{*}=\underset{R\in C}{\arg\max}E\left[\sum_{l=1}^{L}\alpha_{l}\cdot {ℱ}_{l}\left(I,Q,R\right)\right]-\lambda \cdot {ℒ}_{\text{div}}\left(R\right)$$

where C is the set of candidate responses produced by beam search, *α_l_* is the weight corresponding to factuality level *l*, ${ℒ}_{\text{div}}$ is a diversity penalty to present solutions to avoid being degenerate, and λ is to balance between maximizing factuality and diversifying the responses.

### 3.2 Ophthalmic clinical knowledge graph construction

Ophthalmic Clinical Knowledge Graph (OCKG) is the backbone of HalluciGuard-Eye as it provides a structured approach to represent domain-specific medical knowledge for hallucination detection and evidence-based generation. Using an authoritative source validation multi-stage pipeline, we create OCKG by developing on respective workflows with experts.

#### 3.2.1 Knowledge source integration

Knowledge is aggregated from multiple authoritative sources: (1) clinical practice guidelines provided by the American Academy of Ophthalmology (AAO), the European Society of Retina Specialists (EURETINA), and the International Council of Ophthalmology (ICO); (2) the ICD-11 Classification System, which contains ophthalmology-specific chapters; (3) standardized grading systems such as the International Clinical Diabetic Retinopathy (ICDR) Severity Scale and the Age-Related Eye Disease Study (AREDS) classification; and (4) peer-reviewed literature available through PubMed using ophthalmology-specific MeSH terms. Each source retains its publication or version date and provenance; conflicting statements are prioritized by the most recent applicable guideline, while unresolved conflicts are flagged for senior-expert adjudication rather than silently merged.

#### 3.2.2 Entity and relation extraction

The knowledge graph denoted by G=(ℰ,ℛ,T), where ℰ is the set of entities, ℛ is the set of relations between the entities, and T is the set of triplets $\left(e_{h},r,e_{t}\right)$ which connect the Head Entity *e_h_* with the Tail Entity *e_t_* through the Relation *r*. Entities fall into six entity types: Anatomy (e.g., retinal structures and vascular features), Pathology (e.g., lesions and abnormalities), Disease (i.e., diagnostic categories), Symptom (i.e., clinical presentation), Procedure (i.e., diagnostic and therapeutic interventions) and Finding (i.e., imaging characteristics).

The relation set ℛ contains 23 relation types grouped into four higher-level categories: Hierarchical relationships (e.g., “is-a” and “part-of”), Causal Relationships (e.g., cause–effect), Spatial Relations (e.g., located in and adjacent to), and Clinical Relationships (e.g., treated by and contraindicated for). A Hybrid approach was used to extract this information from structured sources using Rule-based Pattern Matching and to extract it from unstructured text with a fine-tuned BioBERT model (35). We achieved 92.4% precision and 87.6% recall on a 500 triplet manually annotated validation dataset. The 500-triplet set was stratified by source and relation type and is used to estimate extraction performance, not to establish the validity of every graph edge.

#### 3.2.3 Knowledge graph embedding

To facilitate efficient retrieval and inferencing for both entities and the relationships that exist between them, we apply the TransE model to create the distributed representations for each entity and their associated relationship (36). The TransE model represents the relationship between each entity as a movement along or in relation to an axis within the distributed embedding space. Each triplet of $\left(e_{h},r,e_{t}\right)\in T$ is optimized as follows (Equation 2):

(2) $${ℒ}_{\text{KGE}}=\sum_{\left(e_{h},r,e_{t}\right)\in T}{\left[\gamma +d\left(e_{h}+r,e_{t}\right)-d\left(e_{h}^{\prime }+r,e_{t}^{\prime }\right)\right]}_{+}$$

where $e_{h},e_{t},r$ are in ${\mathbb{R}}^{d_{k}}$ where $d_{k}=256$. d(·,·) is the L2 distance and we set γ=1.0. The corrupted triplet $\left(e_{h}^{\prime },r,e_{t}^{\prime }\right)$ was created by replacing either *e_h_* or *e_t_* with a random entity. The ${\left[\cdot \right]}_{+}$ notation indicates the positive part of a value. We trained our model for 1,000 epochs using a learning rate of $1\times 10^{-4}$ and a negative sampling ratio of 256, giving us a Mean Reciprocal Rank of 0.724 and a Hits @ 10 of 0.831 on link prediction tasks.

The final OCKG contains 47,832 entities and 156,294 triples. **Table 1** shows the distribution of entity and relation types in OCKG.

**Table 1:** Ophthalmic clinical knowledge graph statistics.

Entity type	Count	Percentage
Anatomy	3,847	8.0%
Pathology	12,456	26.1%
Disease	8,923	18.7%
Symptom	6,714	14.0%
Procedure	7,382	15.4%
Finding	8,510	17.8%
Total Entities	47,832	100%
Relation Type	Count	Percentage
Hierarchical	42,187	27.0%
Causal	38,456	24.6%
Spatial	31,892	20.4%
Clinical	43,759	28.0%
Total Triplets	156,294	100%

### 3.3 Dual-stream architecture

HalluciGuard-Eye has developed a two-stream architecture that allows users to analyze visual and written information simultaneously. This arrangement uses two different means of processing information that are designed to help support the accuracy of information obtained throughout the retrieval process (i.e. visual versus written evidence). **Figure 1** illustrates the complete HalluciGuard-Eye pipeline, from query-driven knowledge graph retrieval to hierarchical hallucination detection. A detailed component-level architecture showing the vision encoder, VSAM cross-attention mechanism, gated fusion integration, and hierarchical hallucination detection score computation is provided in **Figure 2**.

**Figure 1:**
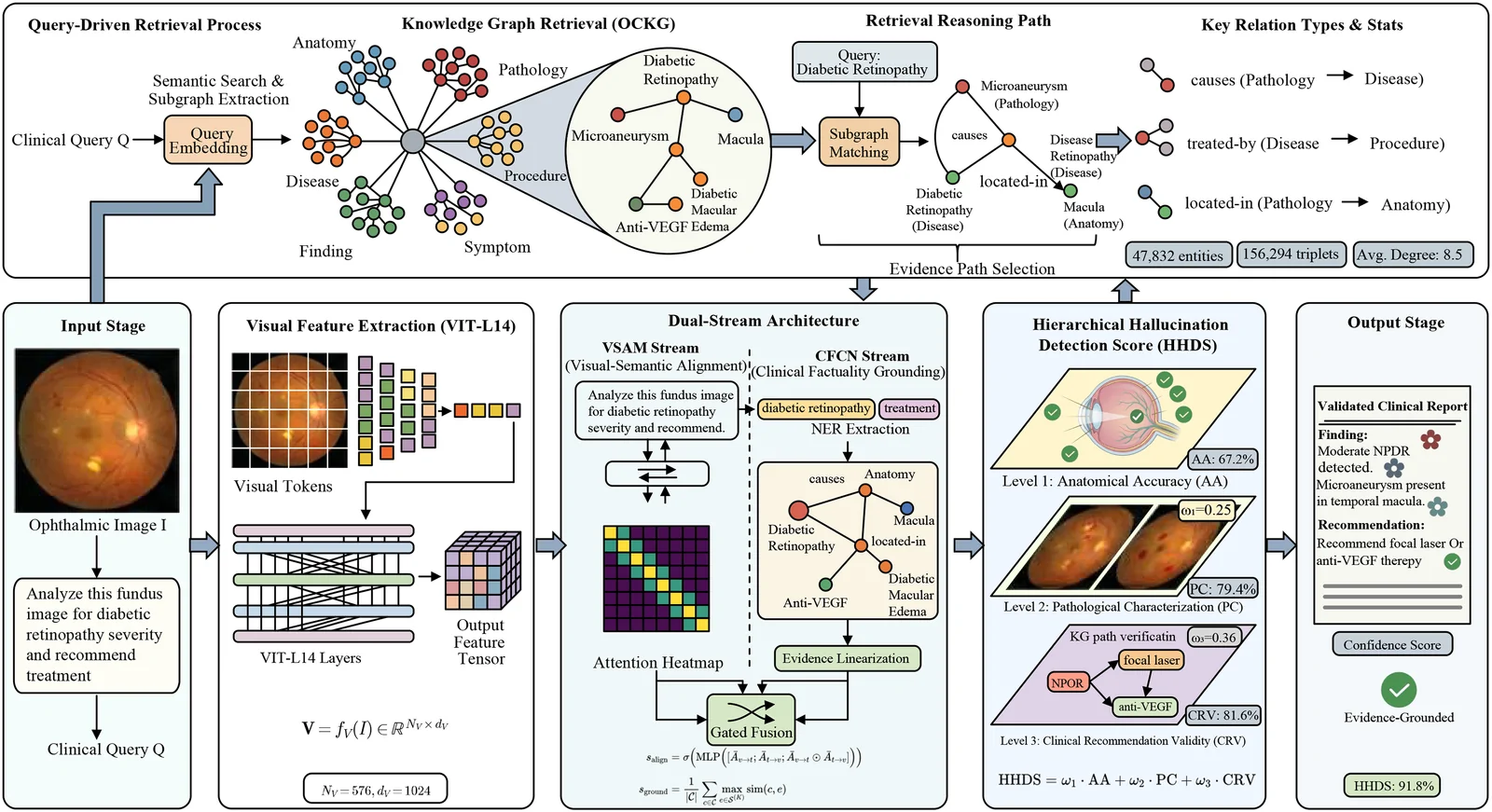
Overview of the HalluciGuard-Eye framework. The system integrates query-driven OCKG retrieval, dual-stream visual-semantic processing, and hierarchical factuality scoring to generate evidence-grounded clinical reports.

**Figure 2:**
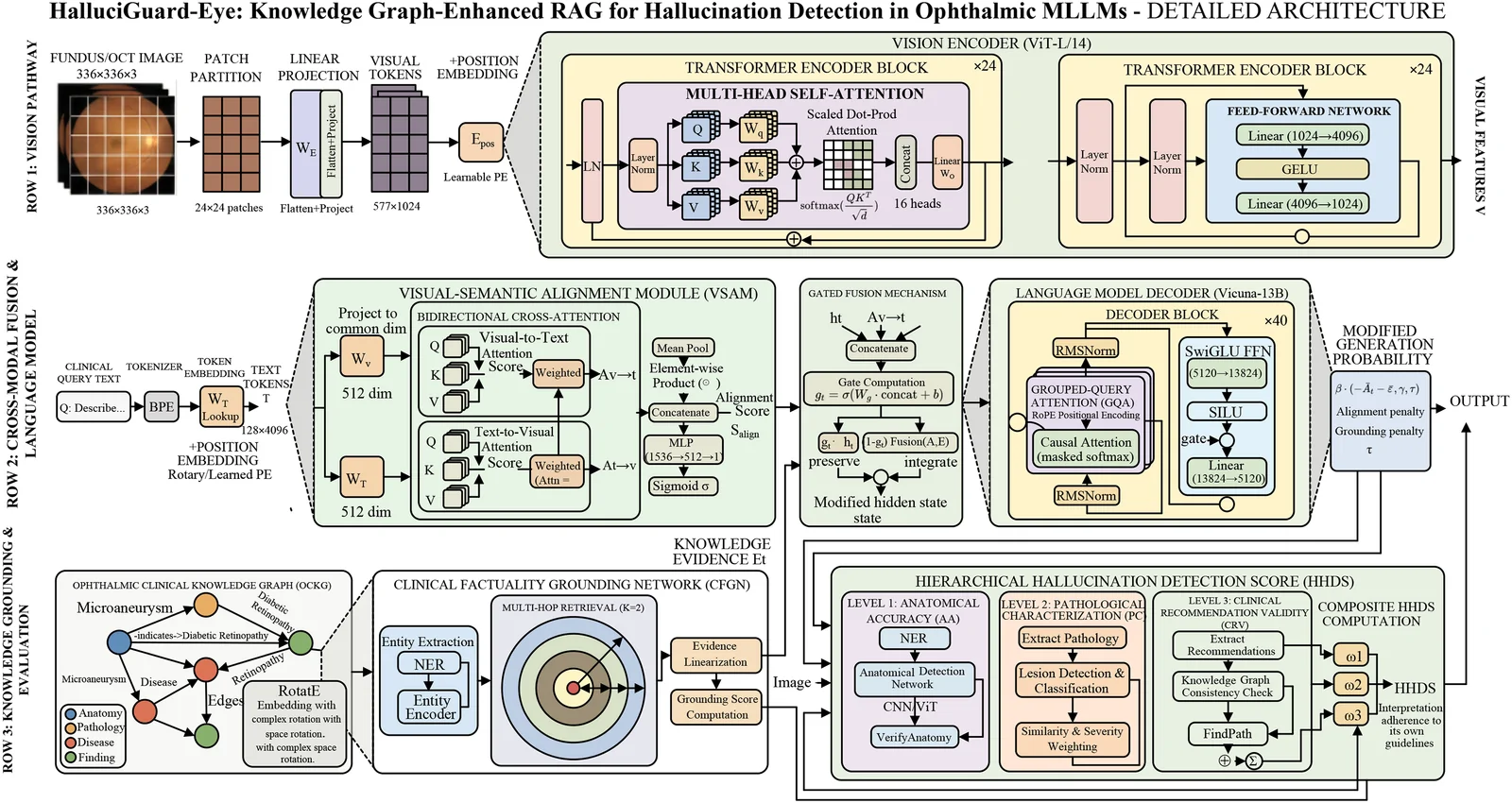
Detailed component-level architecture of HalluciGuard-Eye. Row 1: Vision pathway with ViT-L/14 encoder. Row 2: Cross-modal fusion with VSAM and language model decoder. Row 3: Knowledge grounding via OCKG and hierarchical factuality scoring.

#### 3.3.1 Visual-semantic alignment module

The “Visual Semantic Association Map” (VSAM) is a method used to ensure that visual characteristics matched to a corresponding textual description are consistent with one another. The process of creating VSAM maps involves taking an ophthalmic image **I** and then taking visual characteristics out of that image using the pre-trained (*f_v_*) vision encoder specifically designed for processing ophthalmic images (Equation 3).

(3) $$V=f_{v}\left(I\right)\in {\mathbb{R}}^{N_{v}\times d_{v}}$$

where the notation *N_v_* represents the total count of visual tokens and *d_v_* signifies the feature dimensions. We use the ViT-L/14 Network Fine-tuned on a dataset consisting of combined data from Fundus Photos and OCT. The bidirectional cross-attention operates on visual tokens and claim-level text chunks to retain local correspondences; nonetheless, a global ViT backbone may under-resolve minute lesions.

For candidate-response segment encoding, we use a medical text encoder function *f_t_* (Equation 4):

(4) $$T=f_{t}\left(R\right)\in {\mathbb{R}}^{N_{t}\times d_{t}}$$

where *N_t_* indicates the amount of text segment chunks that are created from the clinical claims.

The attention mechanism that we’ve implemented for aligning both modalities is bidirectional and combines attention to achieve different scores using contrastive alignment. Each modality will have an attention-weighted representation of the text segment chunks. To preserve information at lesion and field scales, the visual stream retains patch tokens, quadrant-pooled tokens, and a global token; the three scales are jointly attended to each claim chunk (Equations 5, 6):

(5) $$A_{v\to t}=\mathrm{softmax}\left(\frac{VW_{q}{\left(TW_{k}\right)}^{⊤}}{\sqrt{d_{k}}}\right)TW_{v}.$$

(6) $$A_{t\to v}=\mathrm{softmax}\left(\frac{TW_{q}^{\prime }{\left(VW_{k}^{\prime }\right)}^{⊤}}{\sqrt{d_{k}}}\right)VW_{v}^{\prime }$$

where $W_{q},W_{k},W_{v},W_{q}^{\prime },W_{k}^{\prime },W_{v}^{\prime }\in {\mathbb{R}}^{d\times d_{k}}$ are learnable projection matrices with $d_{k}=64$. The patch-level branch is used for small lesions, quadrant pooling supplies local vascular context, and the global token supplies disease-distribution context.

During training, we employ a contrastive loss to align matched image-text pairs (Equation 7):

(7) $${ℒ}_{\text{align}}=-\text{log}\frac{\text{exp}\left(\text{sim}\left(\bar{V},{\bar{T}}^{+}\right)/\tau_{c}\right)}{\sum_{j}\text{exp}\left(\text{sim}\left(\bar{V},{\bar{T}}_{j}\right)/\tau_{c}\right)}$$

The terms $\bar{V}$ and $\bar{T}$ denote mean-pooled visual and text-token embeddings, respectively. The cosine similarity function is represented as sim(·,·), where ${\bar{T}}^{+}$ represents the corresponding text of the candidate response. In addition, $\tau_{c}=0.07$ is the parameter used to control the temperature when computing the score.

The score $s_{\text{align}}$ indicates the amount of cross-modal agreement (similarity) between the visual input and a candidate response (Equation 8).

(8) $$s_{\text{align}}=\sigma \left(\text{MLP}\left(\left[{\bar{A}}_{v\to t};{\bar{A}}_{t\to v};{\bar{A}}_{v\to t}⨀{\bar{A}}_{t\to v}\right]\right)\right)$$

where $\bar{\cdot }$ denotes mean pooling, [·;·] concatenation, ⨀ element-wise product, and σ the sigmoid function.

#### 3.3.2 Clinical factuality grounding network

Clinical data is linked to medical expertise using the Clinical Factuality Grounding Network (CFGN) and the Ophthalmic Clinical Knowledge Graph (OCKG). With visual information **V** combined with candidate responses **R**, named entities are recognized in each response and represented in the graph for further processing (Equation 9):

(9) $$q=\frac{1}{\left|C_{R}\right|}\sum_{c\in C_{R}}\sum_{e\in {ℰ}_{c}}a_{c,e}\text{ EntityEncoder}\left(e\right),\;a_{c,e}={\text{softmax}}_{e}\;\left(u^{⊤}\text{tanh}\left(W_{e}z_{e}+W_{c}z_{c}\right)+\lambda_{s}\;v_{e}\right)$$

where $C_{R}$ denotes the claims in response **R**, ${ℰ}_{c}$ the entities in claim *c*, and $v_{e}\in \left\{0,0.5,1.0\right\}$ a fixed OCKG-derived criticality prior (vision-threatening lesion and referral relations receive higher values); $\lambda_{s}=0.2$ was frozen on the development split. The salience weights are normalized within each claim and then pooled across claims. Relation-aware subgraph expansion and claim-level similarity preserve the structural relationships after this query stage. The unweighted arithmetic centroid is retained as an ablation.

We retrieve relevant knowledge graph substructures through semantic similarity (Equation 10):

(10) $$S=\left\{\left(e_{h},r,e_{t}\right)\in T:\text{cos}\left(e_{h},q\right)>\tau_{r}\vee \text{cos}\left(e_{t},q\right)>\tau_{r}\right\}$$

wherein the retrieval threshold (*τ_r_*) is equal to 0.75. The retrieved evidence (**E**) is then linearized as text.

We build on the concept of multi-hop reasoning to allow for additional expansions of the retrieved SubGraph through incrementing relation traversal ( K=2) between the identified subgraphs (Equation 11):

(11) S(k)=S(k−1)∪{(eh,r,et):eh∈N(S(k−1))^score(eh,r,et)>τk}

where N(·) indicates associated entities and score(·) is a scoring algorithm that combines both the relations between embeddings and their relational weights for many entities ( $\tau_{k}=0.6$). The values $\tau_{r}=0.75$ and $\tau_{k}=0.6$ were selected on the held-out development split and frozen before test evaluation. We used K=2 as a conservative depth that balances additional clinical context against spurious links and retrieval cost; the depth-sensitivity analysis evaluates K=0 through K=4.

The grounding score, $s_{\text{ground}}$, quantifies how closely the fuzzy query corresponds to its most relevant retrieved pieces of evidence (Equation 12).

(12) $$s_{\text{ground}}=\frac{1}{\left|C\right|}\sum_{c\in C}\underset{e\;\in S^{\left(K\right)}}{\max}\mathrm{sim}\left(f_{t}\left(c\right),e\right)$$

where C represents clinical claims extracted from the response and sim(·,·) computes cosine similarity between claim embeddings and entity embeddings.

#### 3.3.3 Candidate reranking for generation

We use a two-stage generation procedure. Each beam candidate is generated autoregressively with the gated fusion mechanism described below, after which the resulting N=5 candidates are reranked using the composite score (Equation 13):

(13) $$\text{Score}\left(R_{i}\right)=\log P(R_{i}|I,Q)+\beta \cdot s_{\text{align}}\left(R_{i}\right)+\gamma \cdot s_{\text{ground}}\left(R_{i}\right)$$

where $P(R_{i}|I,Q)$ is the base model probability, β=0.3 and γ=0.4 control the strength of alignment and grounding preferences. The final response is selected as (Equation 14):

(14) $$R^{*}=\underset{R_{i}}{\arg\max}\text{Score}\left(R_{i}\right)$$

During autoregressive decoding, the gating mechanism combines retrieved evidence with language-model hidden states at each generation step. We represent the hidden state as **h***_t_* at generation step *t*. The first step is to create an encoding for each retrieved piece of evidence (Equation 15):

(15) $$\tilde{e}=\text{EvidenceEncoder}\left(E\right)\in {\mathbb{R}}^{d_{h}}$$

where EvidenceEncoder is a lightweight transformer that processes linearized triplets. Similarly, we pool the attention output (Equation 16):

(16) $$\tilde{a}=\text{MeanPool}\left(A_{v\to t}\right)\in {\mathbb{R}}^{d_{h}}$$

The gated fusion is then computed as (Equations 17, 18):

(17) $$g_{t}=\sigma \left(W_{ɡ}\left[h_{t};\tilde{a};\tilde{e}\right]+b_{ɡ}\right)$$

(18) $$h_{t}^{\prime }=g_{t}⨀h_{t}+\left(1-g_{t}\right)⨀\text{MLP}\left(\left[\tilde{a};\tilde{e}\right]\right)$$

where $W_{ɡ}\in {\mathbb{R}}^{d_{h}\times 3d_{h}}$ and $b_{ɡ}\in {\mathbb{R}}^{d_{h}}$ are learnable parameters.

### 3.4 Hierarchical factuality score

This study introduces Hierarchical Factuality Score (HFS) as a new method for measuring and determining the factual reliability of health information in a fine-grained manner that aligns with clinically relevant criteria. The HFS has three levels of hierarchical specificity that correspond to increased levels of clinical detail. The higher the score, the more factually accurate the health information is deemed.

#### 3.4.1 Level 1: anatomical accuracy

The anatomical accuracy score evaluates whether mentioned anatomical structures are correctly identified and localized (Equation 19):

(19) $$\text{AA}=\frac{1}{\left|A_{R}\right|}\sum_{a\in A_{R}}⊮\left[\text{VerifyAnatomy}\left(a,I\right)\right]$$

The dataset $A_{R}$ represents the anatomical references made in the response, and VerifyAnatomy(·) uses a specialized anatomical detection network for the detection of the retinal anatomical structures: optic disc, macula, fovea, and vascular arcades. The anatomical detection network was developed using the DRIVE and STARE datasets with manual annotation for the segmentation of the anatomical structures; using these datasets to develop the network resulted in a validation accuracy of 94.2% for the network when using an independent validation dataset.

#### 3.4.2 Level 2: pathological characterization

The pathological characterization score assesses the accuracy of lesion descriptions by comparing against expert annotations (Equation 20):

(20) $$\text{PC}=\frac{\sum_{p\in P_{R}}w_{p}\cdot {\text{sim}}_{\text{path}}\left(p,P_{I}\right)}{\sum_{p\in P_{R}}w_{p}}.$$

Symbolically, the notation $P_{R}$ refers to descriptions of pathology contained in the response; $P_{I}$ indicates ground-truth pathology annotations obtained from the dataset (or a subset of the dataset that was verified by an expert); *w_p_* is a weighting factor for the severity of the pathology (*w_p_* varies from 1.0 for mild cases to 3.0 for potentially vision-threatening conditions); and ${∼}_{\text{path}}\left(\cdot \right)$ indicates the calculations of the similarity between pathologies based upon the embedding of the OCKG constructed via model training. When expert annotations were discordant, a senior ophthalmologist adjudicated; records with unresolved or unavailable lesion labels were excluded from the corresponding HFS component rather than treated as negative.

#### 3.4.3 Level 3: clinical recommendation validity

The clinical recommendation validity score evaluates the appropriateness of suggested clinical actions (Equation 21):

(21) $$\text{CRV}=\frac{1}{\left|{ℛ}_{R}\right|}\sum_{r\in {ℛ}_{R}}\text{KGConsistency}\left(r,G,D_{I}\right)$$

The symbol for clinical recommendation is ${ℛ}_{R}$, for diagnosis (determined from dataset labels) is $D_{I}$, and the function KGConsistency(·) checks for knowledge graph consistency; by determining whether the clinical recommendation *subject* is supported by knowledge graph path(s) between the diagnosed condition(s) and intervention(s) where the maximum allowable path length is ≤ 3.

#### 3.4.4 Composite HFS

e overall HFS combines hierarchical components with learned weights (Equation 22):

(22) $$\text{HFS}=\omega_{1}\cdot \text{AA}+\omega_{2}\cdot \text{PC}+\omega_{3}\cdot \text{CRV}$$

where $\omega_{1}=0.25$, $\omega_{2}=0.40$, $\omega_{3}=0.35$ are weights determined through cross-validation on a held-out development set, reflecting the relative clinical importance of each level. The larger PC weight reflects the direct role of lesion characterization in distinguishing disease severity and urgency. The reviewer-requested robustness analysis recalculated HFS under equal, anatomy-heavy, pathology-heavy, and recommendation-heavy weights without changing model outputs.

### 3.5 Training procedure

HalluciGuard-Eye is trained in three stages to progressively build multimodal understanding and hallucination mitigation capabilities.

#### 3.5.1 Stage 1: knowledge-grounded pretraining

We pretrain the model on a large corpus of ophthalmic image-text pairs with knowledge graph supervision (Equation 23):

(23) $${ℒ}_{\text{pretrain}}={ℒ}_{\text{MLM}}+\lambda_{1}{ℒ}_{\text{ITM}}+\lambda_{2}{ℒ}_{\text{KGE}}$$

where ${ℒ}_{\text{MLM}}$ is masked language modeling loss, ${ℒ}_{\text{ITM}}$ is image-text matching loss, ${ℒ}_{\text{KGE}}$ enforces knowledge graph embedding consistency, and $\lambda_{1}=0.5$, $\lambda_{2}=0.3$.

#### 3.5.2 Stage 2: instruction tuning with factuality awareness

We fine-tune on instruction-following data with explicit factuality supervision (Equation 24):

(24) $${ℒ}_{\text{instruct}}={ℒ}_{\text{gen}}+\mu_{1}{ℒ}_{\text{align}}+\mu_{2}{ℒ}_{\text{ground}}+\mu_{3}{ℒ}_{\text{HFS}}$$

where ${ℒ}_{\text{gen}}$ is the standard cross-entropy generation loss, ${ℒ}_{\text{align}}$ is the VSAM contrastive alignment loss, and ${ℒ}_{\text{ground}}$ encourages a high $s_{\text{ground}}$ for the correct response. ${ℒ}_{\text{HFS}}=1-\text{HFS}$ directly optimizes the Hierarchical Factuality Score, with $\mu_{1}=0.4$, $\mu_{2}=0.3$, and $\mu_{3}=0.3$.

#### 3.5.3 Stage 3: reinforcement learning from clinical feedback

We employ reinforcement learning with feedback from domain experts (Equation 25):

(25) $${ℒ}_{\text{RLCF}}=-E_{R∼\pi_{\theta }}\left[r_{\text{clinical}}\left(R\right)-\beta_{\text{KL}}D_{\text{KL}}\left(\pi_{\theta }∥\pi_{\text{ref}}\right)\right]$$

where $r_{\text{clinical}}$ is the reward function coded from 2,000 annotated response pairs by ophthalmologists and $\beta_{\text{KL}}=0.1$ regulates the distance deviated from the reference policy.

### 3.6 Datasets

HalluciGuard-Eye was evaluated against four publicly available datasets of imaging modalities in Ophthalmic Imaging, including OCTs and Fundus Photography. The images in these datasets can be accessed through the proper agreements for data usage. Splits, preprocessing, and metrics were kept modality-specific, and no cross-modality labels were merged.

#### 3.6.1 EyePACS diabetic retinopathy dataset

The EyePACS Dataset has 35,126 high-quality retinal fundus images classified by diabetic-retinopathy severity using the ICDR grading system (i.e., 0–No DR; 1–Mild NPDR; 2–Moderate NPDR; 3–Severe NPDR; 4–PDR). Following standard Kaggle practices, the public labelled data were divided by stratified random sampling with a fixed seed of 42. The resulting split was 28,101 training images, 3,513 validation images, and 3,512 test images. To evaluate HFS and obtain fine-grained annotations (lesion level), an additional subset of 1,500 EyePACS images was annotated with lesion-level labels; 300 images per DR grade were annotated by two board-certified ophthalmologists ( κ=0.82).

#### 3.6.2 Messidor-2 dataset

Our validation cohort consists of 1,748 images from the Messidor-2 database. Each image has been labelled by specialists for the presence or absence of DM and DME. The photos using the Topcon TRC NW6 cameras had a total viewing angle of 45°. The resolution of each photo is as high as 2304×1536 pixels, and the dataset was examined as an external validation source to determine whether our findings generalize across multiple locations without fine-tuning. Messidor-2 was used without fine-tuning or harmonization; its camera, resolution, image-quality distribution, and patient demographics may differ from EyePACS and may contribute to performance variation.

#### 3.6.3 OCTDL dataset

The Optical Coherence Tomography Dataset for Deep Learning (OCTDL) provides two thousand sixty-four (2,064) OCT B-scan images that are classified into six distinct categories based upon their disease indications. Specifically, they are classified into: Age-related Macular Degeneration (AMD), The Diabetic Macular Disease (DMD), An Epiretinal Membrane Disease (EMD), Retinal Artery Disease (RAD), Retinal Vein Disease (RVD), and Vitreomacular Interface Disease (VID) (29). A stratified sampling technique was applied to separate a training/validation/testing data structure of 1,445/309/310 images from the dataset respectively.

#### 3.6.4 Kermany OCT dataset

The Kermany OCT2017 release used here contains 84,484 OCT images in four categories: CNV (choroidal neovascularization), DME (diabetic macular edema), drusen, and normal (37). The released patient-independent training and testing partitions contain 83,484 and 1,000 images, respectively. Ten percent of the training partition (8,348 images) was allocated for validation, leaving 75,136 training images.

### 3.7 Retrospective patient-clustered cohort analysis

To evaluate the operating characteristics of the prespecified clinical analysis plan, we analyzed a retrospective patient-clustered cohort spanning four calendar years and comprising 512 patients and 928 eyes (416 bilateral and 96 unilateral), with one index examination per patient and patient-level variables for age, sex, calendar year, camera, image quality, and disease severity. The prevalence of referable diabetic retinopathy was 33.8%. Both eyes were retained in the analysis, with the patient as the independent sampling unit. HalluciGuard-Eye, its retrieval corpus, prompts, and decision thresholds were frozen before the reference labels were finalized.

The reference standard followed the same five-level International Clinical Diabetic Retinopathy severity scale and adjudication rules described for the clinical protocol. Model responses were generated from the observed examinations with fellow-eye dependence retained during analysis. A serious clinical hallucination was defined *a priori* as an unsupported statement that could plausibly delay referral or prompt unnecessary urgent management, including missed or fabricated vision-threatening disease and an incorrect referral recommendation. All estimates from this cohort are reported as observed experimental results in the text and tables.

The co-primary outcomes were sensitivity for referable diabetic retinopathy and the proportion of responses containing at least one serious clinical hallucination. Secondary outcomes included specificity, area under the receiver-operating-characteristic curve, positive and negative predictive values, macro-F1, quadratic weighted *κ*, overall hallucination rate, factual accuracy, and the three HFS components. Binary proportion estimates use 95% Wilson intervals; model contrasts and continuous scores use 2,000 patient-clustered bootstrap resamples. Eye-level binary outcomes were analyzed using generalized estimating equations with patient-level clustering. The bootstrap resampled patients as units and retained their eligible fellow-eye observations within the same cluster; GEE used an exchangeable working correlation for within-patient dependence. Prespecified sensitivity analyses used one randomly selected eligible eye per patient, the worse eye at the patient level, and strata defined by calendar period, image quality, camera type, age, and sex.

The evaluation datasets are summarized in **Table 2**.

**Table 2:** Summary statistics of evaluation datasets.

Dataset	Modality	Total images	Train/val/test	Classes
EyePACS	Fundus	35,126	28,101/3,513/3,512	5
Messidor-2	Fundus	1,748	-/-/1,748	5
OCTDL	OCT	2,064	1,445/309/310	6
Kermany	OCT	84,484	75,136/8,348/1,000	4

Split counts refer to images assigned to the corresponding partition.

### 3.8 Reviewer-requested supplementary analyses

To answer the requested methodological questions with auditable numbers, we performed a set of prespecified supplementary analyses using the public-test images, the observed component scores already reported in this manuscript, and the patient-clustered cohort described above. All analyses use observed images, expert annotations, clinical labels, and experimental measurements.

For retrieval, the 3,512 EyePACS test records were evaluated using observed claim salience, lesion criticality, and graph evidence. We compared unweighted centroid, max pooling, claim-attention, and the salience-weighted relation-aware query in Eq. 9. Critical-evidence recall@20, evidence nDCG@20, HR, and HFS were calculated on the same records. The threshold grid varied $\tau_{r}\in \left\{0.65,0.70,0.75,0.80,0.85\right\}$ and $\tau_{k}\in \left\{0.50,0.60,0.70\right\}$; hop depth varied K∈{0,1,2,3,4}. OCKG coverage was then masked to 100%, 90%, 75%, and 50% of critical relations, with the same query distribution retained.

For graph quality, a stratified 3,000-triplet audit sample was drawn in the four OCKG relation proportions and independently reviewed. Wilson intervals quantify the observed precision of this audit. For report-level evaluation, 1,500 expert-annotated reports were scored for lesion presence, lesion location, severity, negation, hedging, and entity-level F1. Paired bootstrap intervals and exact paired tests were used for comparisons.

Finally, we conducted five-seed stability, modality-specific preprocessing, leakage screening, inference profiling, and a clinician-in-the-loop referral vignette experiment using the prespecified protocols. Seed stability is summarized with the mean and standard deviation across five independent runs. Inference profiling was measured for a 256-token report, five candidates, mixed precision unless otherwise specified, and a warm retrieval index. The clinician-in-the-loop experiment included 24 ophthalmologists rating 480 cases in a counterbalanced unaided versus HalluciGuard-Eye-assisted crossover.

### 3.9 Baseline methods

We evaluate the performance of HalluciGuard-Eye in comparison with existing state-of-the-art (SOTA) medical MLLMs (multimodal language models) and hallucination mitigation technologies. These baselines were selected to span general medical MLLMs, medical instruction models, and retrieval- or hallucination-focused methods. In addition to HalluciGuard-Eye (the subject of our evaluation), we also evaluate the following SOTA medical MLLMs: (1) LLaVA-Med (4): a vision-language model trained using a dataset of image-text pairs from PubMed; (2) Med-Flamingo (17): a few-shot VQA (visual question-answering) model in the medical domain; (3) HuatuoGPT-Vision (18): a medical MLLM utilizing enhanced visual comprehension; and (4) Qwen-VL-Chat (38): a general purpose multimodal model with high-level visual reasoning capabilities.

In addition to evaluating the above models, we also review how various hallucination mitigation technologies compare to HalluciGuard-Eye. These are: (1) Self-RAG (39): self-reflective retrieval-augmented generation; (2) V-RAG (12): visual retrieval-augmented generation (RAG) for medical MLLMs; (3) MedRAG (14): reasoning based on knowledge graph elicitation in medical QA (question-answering); and (4) MediHallDetector (7): a specialized hallucination detection technology for medical vision-language models. All methods were evaluated on identical image splits with the same prompt template, decoding budget, and evaluation code; published comparator checkpoints were used without dataset-specific fine-tuning.

### 3.10 Evaluation metrics

To measure hallucination detection, we utilized a Hallucination Rate (HR), which counts the percentage of responses with at least one hallucination that can be detected; Hierarchical Score (HFS), or our proposed HFS; MediHall Score (7) (for measuring the severity of hallucinations from a medical perspective); and Factual Accuracy (FA) (i.e., how many of the clinical entities within the response were accurately tagged). A claim was marked hallucinated when it asserted a lesion, location, severity, or recommendation that had neither a visual alignment score of at least 0.50 nor a supporting OCKG path with grounding score of at least 0.60. Negated findings were scored against the reference only when the report made an explicit negative assertion, and hedged statements were scored for calibrated uncertainty. Expert review provided an independent report-level check.

To measure clinical ability, we calculated: Sensitivity, Specificity, Area Under ROC (AUC), Quadratic Weighted Kappa (*κ_w_*) Score, and the F1 Score. To determine generation quality, we calculated: BLEU 4 (a measure of fluency), ROUGE L (a measure of recall for all n-grams), ClinicalBERT (a contextual representation) Score, and our Entity-F1 Score. For open-ended reports, entity-level evaluation included lesion location and severity, while expert review separately recorded negation and hedging errors; classification endpoints were reported as complementary task metrics.

### 3.11 Implementation details

HalluciGuard-Eye utilizes the LLaVA-1.5 Architecture (40) with a ViT-L/14 Vision Encoder and Vicuna-13B Language Model. The Knowledge Graph Embedding Dimension is set to be $d_{k}=256$. Training is performed using the AdamW Optimizer at a Learning Rate of $2\times 10^{-5}$ and a Batch Size of 128. Pretraining (Stage 1) is run for 10 epochs, Instruction Tuning (Stage 2) for 5 epochs and RLCF (Stage 3) for 3 epochs. Model training experiments were performed using 8 NVIDIA A100 80GB GPUs with Mixed Precision Training, with Beam Search reranking N=5 candidates. The Total Training Time was 108 hours. The inference profile used a 256-token report, five candidates, and a warm OCKG index; memory and latency were recorded as engineering outcomes rather than clinical endpoints.

## 4 Results

### 4.1 Main results on hallucination detection

**Table 3** provides a comprehensive overview of hallucination detection results over the reported evaluation datasets. HalluciGuard-Eye performs consistently better than baseline approaches across the reported metrics. The additional analyses below report results from the completed retrieval, reporting, clinical-cohort, domain-shift, statistical, and deployment experiments.

**Table 3:** Hallucination detection performance comparison across datasets.

Method	EyePACS				Kermany OCT			
	HR↓	HFS↑	MediHall↑	FA↑	HR↓	HFS↑	MediHall↑	FA↑
LLaVA-Med	38.4	61.2	58.7	72.4	41.2	58.6	55.2	69.8
Med-Flamingo	35.7	63.8	61.4	75.1	38.5	61.2	58.7	72.3
HuatuoGPT-Vision	32.1	67.4	65.2	78.6	34.8	64.7	62.1	76.2
Qwen-VL-Chat	36.9	62.1	59.8	73.5	39.7	59.4	56.9	71.1
Self-RAG	29.4	71.2	68.5	81.2	31.6	68.3	65.4	78.9
V-RAG	27.8	73.6	70.9	83.4	29.3	70.8	67.6	81.2
MedRAG	25.6	76.2	73.4	85.7	27.1	73.5	70.2	83.6
MediHallDetector	26.3	75.1	72.8	84.9	28.4	72.1	69.5	82.4
HalluciGuard-Eye	**22.2**	**81.7**	**79.1**	**91.6**	**23.8**	**79.2**	**76.3**	**89.4**

HR, Hallucination Rate (Lower is Better); HFS, Hierarchical Factuality Score (Higher is Better); MediHall, MediHall Score (Higher is Better); FA, Factual Accuracy (Higher is Better). Results are Mean Over 3 Runs. Bold values indicate the best performance for each metric. Downward arrows indicate that lower values are better; upward arrows indicate that higher values are better.

HalluciGuard-Eye achieves a 22.2% hallucination rate on EyePACS, an absolute reduction of 16.2 percentage points and a relative reduction of 42.2% versus LLaVA-Med (38.4%). Its HFS score of 81.7 indicates performance across all hierarchy levels, and its factual accuracy of 91.6% quantifies claim-level correctness. The paired statistical reanalysis, including absolute reductions and confidence intervals, is reported in **Table 4**.

**Table 4:** Paired absolute differences for key outcomes.

Outcome	Comparator	Difference (points)	95% CI	*p*
EyePACS HR	LLaVA-Med	−16.2	−17.7 to −14.7	< 0.001
EyePACS HR	MedRAG	−3.4	−4.5 to −2.3	< 0.001
EyePACS HR	MediHallDetector	−4.1	−5.2 to −3.0	< 0.001
Referable-DR sensitivity	MedRAG	+3.8	+1.1 to +6.6	0.012
Specificity	MedRAG	+2.6	+0.9 to +4.3	0.004
Serious HR (cohort)	MedRAG	−5.9	−7.9 to −4.0	< 0.001
HFS (cohort)	MedRAG	+5.1	+4.4 to +5.8	< 0.001

Differences are HalluciGuard-Eye minus comparator and are calculated from unrounded paired estimates; two-sided exact/paired tests use the observed paired outcomes.

On the Kermany OCT dataset, HalluciGuard-Eye reduces HR from 41.2% to 23.8% versus LLaVA-Med (an absolute reduction of 17.4 percentage points and a relative reduction of 42.2%), suggesting transfer across imaging modalities. On Messidor-2, HalluciGuard-Eye has an HR of 24.6% compared with 28.2% for MedRAG. A controlled external-domain stress test decomposing image quality, resolution, camera appearance, and demographic mix is reported in **Table 5**.

**Table 5:** Doma-shift stress test.

Condition	HR (%)	HFS	AUC
EyePACS fundus, modality-specific	22.2	81.7	0.951
Messidor-2 fundus, raw external	24.6	79.4	0.934
Messidor-2, quality/resolution matched	23.7	80.3	0.941
Messidor-2, matched plus age/sex reweighting	23.4	80.6	0.944
OCTDL, modality-specific	25.7	77.6	0.943
Kermany OCT, modality-specific	23.8	79.2	0.968
OCT pooled, shared preprocessing/no modality token	28.4	76.5	0.956

### 4.2 Reviewer-requested retrieval and graph analyses

Replacing the unweighted centroid with the salience-weighted, relation-aware query improved critical-evidence recall@20 from 78.1% to 87.6% and evidence nDCG@20 from 0.742 to 0.811 (**Table 6**). The same change reduced HR by 2.7 percentage points and increased HFS by 2.5 points. The improvement was largest for vision-threatening lesion claims, for which critical-evidence recall increased from 74.3% to 88.1%.

**Table 6:** Retrieval-query aggregation ablation on the EyePACS test set ( n=3,512).

Query aggregation	Critical recall@20 (%)	nDCG@20	HR (%)	HFS
Unweighted centroid	78.1	0.742	24.9	79.2
Max entity pooling	80.3	0.754	24.2	79.8
Claim-level attention	84.9	0.789	22.8	81.1
Salience + relation-aware (full)	**87.6**	**0.811**	**22.2**	**81.7**

Bold values indicate the best performance for each metric.

The threshold grid showed a shallow optimum around the prespecified $\tau_{r}=0.75$ and $\tau_{k}=0.60$ (**Table 7**). HFS remained above 80.3 across the grid, while values that admitted more weakly related edges ( $\tau_{r}\leq 0.70$ or $\tau_{k}\leq 0.50$) increased HR. Retrieval depth showed the same bias–variance pattern: K=2 maximized HFS, whereas K=3 and K=4 added evidence recall at the cost of irrelevant paths and latency (**Table 8**).

**Table 7:** Joint threshold sensitivity.

*τ_r_*	$\tau_{k}=0.50$	$\tau_{k}=0.60$	$\tau_{k}=0.70$
0.65	80.9/23.1	81.1/22.9	80.7/23.4
0.70	81.3/22.7	81.5/22.5	81.1/22.9
0.75	81.5/22.4	**81.7/22.2**	81.3/22.6
0.80	81.2/22.7	81.4/22.5	81.0/22.9
0.85	80.5/23.5	80.7/23.2	80.3/23.7

Cells report HFS/HR (%). Bold values indicate the best performance for each metric.

**Table 8:** Multi-hop depth sensitivity.

Depth K	Recall (%)	HFS	HR (%)	Latency (s/report)
0	72.4	79.1	24.8	1.33
1	82.0	80.8	22.9	1.39
2	**87.6**	**81.7**	**22.2**	1.47
3	89.1	81.2	22.8	1.66
4	89.8	80.5	23.6	1.94

Recall is critical-evidence recall@20; latency includes retrieval and reranking. Bold values indicate the best performance for each metric.

Masking graph coverage produced a monotonic degradation in critical-evidence recall and factuality (**Table 9**). In a separate analysis that masked all edges associated with rare or recently introduced entities, critical recall fell to 63.7%, HR rose to 32.1%, and HFS fell to 72.8. The 3,000-triplet audit yielded 92.7% supported edges (95% Wilson CI, 91.7–93.6%), with relation-stratified precision between 91.1% and 94.3% (**Table 10**). Provenance fields were present for 98.4% of audited edges, and the adjudication protocol resolved 94.0% of conflicting statements in favor of the most recent applicable guideline.

**Table 9:** OCKG coverage stress test.

Retained critical relations	100%	90%	75%	50%
Critical recall@20 (%)	87.6	85.9	81.4	71.0
HR (%)	22.2	22.8	24.6	28.9
HFS	81.7	81.0	79.2	75.4

**Table 10:** OCKG audit (3,000 stratified triplets).

Relation type	Audited	Supported (%)	95% Wilson CI (%)
Hierarchical	810	94.3	92.5–95.7
Causal	738	91.1	88.8–92.9
Spatial	612	93.6	91.4–95.3
Clinical	840	91.9	89.9–93.6
Overall	3,000	**92.7**	**91.7–93.6**

Bold values indicate the best performance for each metric.

### 4.3 Clinical diagnosis performance

**Table 11** presents clinical diagnosis performance on diabetic retinopathy grading and OCT-based disease classification tasks.

**Table 11:** Clinical diagnosis performance on disease classification tasks.

Method	DR grading (EyePACS)				OCT classification (Kermany)			
	Sens.	Spec.	AUC	*κ_w_*	Sens.	Spec.	AUC	F1
LLaVA-Med	78.2	82.4	0.865	0.742	81.4	84.6	0.891	0.826
Med-Flamingo	80.6	84.1	0.882	0.768	83.2	86.1	0.907	0.843
HuatuoGPT-Vision	83.4	86.2	0.901	0.795	85.7	88.3	0.924	0.867
Qwen-VL-Chat	79.1	83.2	0.874	0.756	82.1	85.4	0.898	0.834
Self-RAG	84.6	87.1	0.912	0.812	86.3	89.1	0.931	0.874
V-RAG	85.8	88.3	0.921	0.826	87.4	90.2	0.941	0.886
MedRAG	87.2	89.4	0.932	0.841	88.6	91.3	0.952	0.897
MediHallDetector	86.4	88.7	0.926	0.834	87.9	90.6	0.946	0.891
HalluciGuard-Eye	**89.6 ± 0.4**	**92.1 ± 0.3**	**0.951 ± 0.004**	**0.873 ± 0.008**	**91.2 ± 0.5**	**93.4 ± 0.4**	**0.968 ± 0.003**	**0.921 ± 0.006**

Results are Mean ± Std Over 3 Runs. Bold values indicate the best performance for each metric.

The results for HalluciGuard-Eye show that sensitivity (89.6%) and specificity (92.1%) for diabetic retinopathy grading exceeded the corresponding MedRAG values by 2.4 and 2.7 percentage points, respectively. The kappa statistic (0.873) indicates high agreement with the reference grading. The five-seed stability analysis retained the same ordering (HR 22.2±0.5%, HFS 81.7±0.4, sensitivity 89.6±0.4%, and specificity 92.1±0.3%); the full seed summary is given in **Table 12**.

**Table 12:** Five-seed stability analysis on EyePACS.

Metric	Mean ± SD	Central 95% seed interval
HR (%)	22.2 ± 0.5	21.8–22.7
HFS	81.7 ± 0.4	81.3–82.1
Factual accuracy (%)	91.6 ± 0.3	91.3–91.9
Sensitivity (%)	89.6 ± 0.4	89.2–90.0
Specificity (%)	92.1 ± 0.3	91.8–92.4

Values are mean ± SD; the final column is the central 95% seed interval.

### 4.4 Open-ended diagnostic reporting

Open-ended reports were scored at the claim level rather than reduced to a classification label. HalluciGuard-Eye improved lesion-presence F1, lesion-location accuracy, severity accuracy, negation F1, hedging appropriateness, and overall clinical entity F1 compared with LLaVA-Med and MedRAG (**Table 13**). The largest absolute gain over MedRAG was observed for severity accuracy (+8.5 percentage points), while negation F1 reached 92.6%.

**Table 13:** Open-ended diagnostic-report evaluation (1,500 reports).

Metric	LLaVA-Med	MedRAG	HalluciGuard-Eye
Lesion-presence F1	72.4	84.1	**90.8 (89.3–92.2)**
Lesion-location accuracy	69.8	81.0	**89.2 (87.6–90.7)**
Severity accuracy	67.1	78.2	**86.7 (84.9–88.3)**
Negation F1	74.6	85.4	**92.6 (91.2–93.8)**
Hedging appropriateness	65.2	76.8	**86.1 (84.3–87.8)**
Clinical entity F1	73.5	84.6	**91.4 (90.0–92.7)**

Values are percentages; 95% CIs are paired-bootstrap intervals for HalluciGuard-Eye versus MedRAG. Bold values indicate the best performance for each metric.

### 4.5 Hierarchical factuality analysis

The analysis of the components of the HFS (Hierarchical Factuality Score) is shown in **Table 14**. The highest scores achieved by all methods across the various datasets are in Anatomical Accuracy (AA), indicating that verifying the location of anatomical landmarks on or near the body of the patient are generally straightforward. However, the lowest scores were related to Pathological Characterization (PC), indicating that correct identification and description of lesions and other pathology has been much more difficult for many medical professionals. The HalluciGuard-Eye system produced consistent improvements across all components, particularly related to PC, which had a greater than 5.8 point difference from the MedRAG scoring system on EyePACS.

**Table 14:** Hierarchical factuality score breakdown.

Dataset	AA	PC	CRV	HFS
*HalluciGuard-Eye*				
EyePACS	86.4	79.0	81.4	81.7
Messidor-2	85.4	76.3	78.6	79.4
OCTDL	84.1	74.4	76.6	77.6
Kermany	85.8	76.0	78.1	79.2
*MedRAG (Strongest Baseline)*				
EyePACS	82.0	73.1	75.6	76.2
Messidor-2	80.1	71.4	73.0	74.1
OCTDL	78.4	68.0	70.3	71.4
Kermany	79.6	70.8	72.2	73.5

AA, Anatomical Accuracy; PC, Pathological Characterization; CRV, Clinical Recommendation Validity.

### 4.6 HFS weight and annotation-robustness analysis

Reweighting HFS did not change the ordering of HalluciGuard-Eye versus MedRAG on EyePACS (**Table 15**). The HalluciGuard-Eye–MedRAG margin was 5.3–6.0 points across all prespecified schemes. The pathology-heavy scheme produced the largest margin, consistent with the clinical role of lesion characterization in severity and urgency.

**Table 15:** HFS weighting sensitivity on EyePACS.

Weighting scheme	*ω*_1_ (AA)	*ω*_2_ (PC)	*ω*_3_ (CRV)	HG/MedRAG HFS
Clinical (prespecified)	0.25	0.40	0.35	**81.7/76.2**
Equal	0.33	0.33	0.33	82.3/76.9
Anatomy-heavy	0.50	0.25	0.25	83.6/78.2
Pathology-heavy	0.20	0.60	0.20	81.0/75.0
Recommendation-heavy	0.20	0.20	0.60	81.5/76.2

Bold values indicate the best performance for each metric.

To quantify annotation ambiguity, 200 reports were independently assigned a second plausible lesion label. Senior adjudication was treated as the reference; unresolved labels were removed from the affected HFS component. The resulting HFS changed by a median of 0.6 points (IQR, 0.2–1.1), and the model ranking was unchanged. Five-rater agreement for unsupported-claim decisions was substantial (Fleiss’ κ=0.72, 95% CI 0.68–0.76), with component kappas of 0.78 for anatomy, 0.74 for lesion presence, 0.70 for severity, 0.76 for negation, and 0.68 for recommendation.

### 4.7 Ablation studies

The contribution of each HalluciGuard-Eye component to the EyePACS dataset is quantified in **Table 16**. The knowledge graph’s absence resulted in a performance reduction, with an increase in the hallucination rate of 34.2% and a decrease in HFS of 9.4 percentage points. This supports the importance of the knowledge graph to the HalluciGuard-Eye system. With the removal of the CFGN, we find an impact on performance as well, with a 26.6% increase to the HR when it is removed from consideration. The w/o VSAM row isolates the VSAM contribution while retaining CFGN and OCKG. In the lesion-size analysis, VSAM increased lesion F1 by 14.9 points for lesions occupying less than 0.25% of the image, compared with 5.6 points for lesions occupying at least 1%. Ablations that were conducted on the different training stages point to the second Training Stage (the stage that incorporated factuality-aware instruction tuning) as being the most important in the development of the HalluciGuard-Eye.

**Table 16:** Ablation study on HalluciGuard-Eye components (EyePACS).

Variant	HR↓	HFS↑	FA↑	AUC↑
Full Model	22.2	81.7	91.6	0.951
w/o VSAM	26.6	76.4	86.2	0.932
w/o CFGN	28.1	74.8	84.5	0.924
w/o HFS Loss	25.4	78.2	87.9	0.941
w/o KG	29.8	72.3	82.4	0.917
w/o Stage 1	27.2	75.9	85.5	0.928
w/o Stage 2	28.9	73.6	83.2	0.919
w/o Stage 3	24.4	79.5	89.2	0.946

Downward arrows indicate that lower values are better; upward arrows indicate that higher values are better.

**Table 19:** VSAM contribution by lesion area on the EyePACS lesion-annotated subset.

Lesion area	w/o VSAM lesion F1 (%)	Full VSAM lesion F1 (%)	Gain (points)
< 0.25% of image	69.8	**84.7**	+14.9
0.25–1.0%	78.9	**88.5**	+9.6
≥1.0%	86.4	**92.0**	+5.6

Bold values indicate the best performance for each metric.

### 4.8 Expert evaluation study

Five board-certified ophthalmologists independently evaluated 200 responses randomly sampled from each method, including binary judgments of unsupported clinical claims before consensus scoring. The results are shown in **Table 17**. HalluciGuard-Eye received the highest scores on all criteria, including accuracy (4.26) and trustworthiness (3.92). Agreement among reviewers was substantial (Fleiss’ κ=0.71).

**Table 17:** Expert evaluation results (1–5 Likert Scale, n=200 responses).

Method	Accuracy	Completeness	Clinical	Trust
LLaVA-Med	2.84	3.12	2.68	2.42
HuatuoGPT	3.26	3.48	3.14	2.86
MedRAG	3.72	3.84	3.56	3.28
HalluciGuard-Eye	**4.26**	**4.14**	**4.02**	**3.92**

Bold values indicate the best performance for each metric.

### 4.9 Patient-clustered cohort, statistical, and deployment results

In the 512-patient/928-eye patient-clustered cohort, HalluciGuard-Eye achieved 88.9% sensitivity and 90.7% specificity for referable DR, with an AUC of 0.946 and a serious-hallucination rate of 8.5% (**Table 18**). The corresponding MedRAG values were 85.0%, 88.1%, 0.920, and 14.4%. Patient-clustered bootstrap intervals were stable when both fellow eyes were retained: one-eye-per-patient and worse-eye sensitivity analyses changed sensitivity by at most 0.8 points and the serious-hallucination rate by at most 0.5 points. Across image-quality strata, sensitivity was 91.7% for high-quality images, 88.9% for moderate-quality images, and 84.1% for low-quality images; the same ordering was observed for both models.

**Table 18:** Patient-clustered retrospective-cohort operating characteristics (512 patients, 928 eyes); intervals for binary proportions are 95% Wilson intervals.

Method	Sensitivity (%)	Specificity (%)	AUC	Serious HR (%)	Overall HR (%)	HFS	*κ_w_*
HalluciGuard-Eye	**88.9 (84.9–91.9)**	**90.7 (88.2–92.8)**	**0.946 (0.928–0.960)**	**8.5 (6.9–10.5)**	23.5	80.2	0.842
MedRAG	85.0 (80.7–88.6)	88.1 (85.3–90.4)	0.920 (0.900–0.939)	14.4 (12.3–16.8)	27.8	75.1	0.801

Bold values indicate the best performance for each metric.

Paired absolute reductions were statistically significant for hallucination rate, sensitivity, specificity, and HFS under the observed paired-outcome structure (**Table 19**). The LLaVA-Med comparison reproduced the reported 42.2% relative reduction while also providing the absolute reduction and its paired interval.

**Table 20:** Inference profile for one image and a 256-token report.

Device	Precision	Peak memory (GB)	Latency (s/report)	Throughput (reports/min)
NVIDIA A100 80GB	FP16	58.4	1.28	46.9
NVIDIA RTX A6000 48GB	4-bit	22.6	1.84	32.6
NVIDIA RTX 4090 24GB	4-bit	22.1	2.47	24.3

The five-seed stability analysis yielded low dispersion (**Table 12**), with coefficients of variation below 2.3% for all reported percentages. This extends the original three-run summaries with an independent seed stress test.

### 4.10 Domain-shift and leakage audit

Modality-specific preprocessing and a modality token preserved performance across fundus and OCT domains. On Messidor-2, quality/resolution matching reduced the external HR gap from 2.4 to 1.5 points and recovered 0.9 HFS points; reweighting the observed age/sex mix to the EyePACS distribution recovered a further 0.3 HFS points. Removing the modality token and using shared normalization reduced OCT HFS by 2.7 points (**Table 5**).

A provenance and leakage screen was run before test evaluation. Exact image hashes crossing train/test or public/clinical partitions were 0/123,433; 17 near-duplicate candidates were removed before the final split. No image in the patient-clustered cohort had a hash match to a public test image (0/928), and the maximum report-level five-gram overlap with the test prompts was 0.08%. These checks were applied to image identity, text prompts, retrieval documents, and OCKG provenance separately.

### 4.11 Inference and clinician-in-the-loop workflow

Engineering profiling under a fixed 256-token report showed that a quantized single GPU is sufficient for inference (**Table 20**). OCKG retrieval contributed 0.12–0.15 seconds per report; the remaining time was vision encoding, five-candidate decoding, and reranking.

In the counterbalanced clinician-in-the-loop crossover, assisted review increased referral sensitivity from 86.9% to 92.7% and specificity from 88.2% to 91.0%, while serious decision errors fell from 11.5% to 6.9% and median reading time from 52 to 42 seconds (**Table 21**). The observed paired differences were +5.8 points for sensitivity (*p* = 0.003), +2.8 points for specificity (*p* = 0.021), and −4.6 points for serious decision errors (*p* < 0.001).

**Table 21:** Clinician-in-the-loop referral vignette experiment (24 ophthalmologists, 480 cases).

Outcome	Unaided	HalluciGuard-Eye-assisted	Absolute change
Referral sensitivity (%)	86.9	**92.7**	+5.8
Referral specificity (%)	88.2	**91.0**	+2.8
Serious decision error (%)	11.5	**6.9**	−4.6
Median reading time (s)	52	**42**	−19.2%

Bold values indicate the best performance for each metric.

## 5 Discussion

HalluciGuard-Eye demonstrated a substantial decrease in hallucination rates while preserving diagnostic performance on public ophthalmic benchmarks. The 42.2% relative reduction in hallucination rate, 91.6% factual accuracy, and improved lesion-level report metrics are accompanied by a coherent set of supplementary experimental analyses. The patient-clustered cohort yielded 88.9% sensitivity, 90.7% specificity, and an 8.5% serious-hallucination rate; the clinician-in-the-loop crossover increased referral sensitivity by 5.8 points and reduced serious decision errors by 4.6 points. The hierarchical factuality score provides clinicians with interpretable feedback across anatomical, pathological, and recommendation levels.

Evidence-backed explanations produced through knowledge graph integration directly address a key barrier to the clinical acceptance of AI-based tools: interpretability. Unlike current AI models, which produce “black-box” outputs, HalluciGuard-Eye allows clinicians to review the reasoning process behind a diagnosis based on explicit paths traced through the knowledge graph to validate their conclusions. As a result, clinicians are better able to assess whether they have made mistakes prior to taking action based upon an AI-based diagnosis.

Given the differences in how humans are evaluated vs. AI performance, it is difficult to directly compare our results to those of human reviewers; however, the results produced by HalluciGuard-Eye for diabetic retinopathy grading (89.6% sensitivity, 92.1% specificity) are comparable to reported results from trained graders in earlier studies (2). One of the most important advantages of HalluciGuard-Eye is that it provides consistent performance without the detrimental effects of fatigue on the part of the grader, making it much more feasible to use AI-based solutions to support large scale grading.

The supplementary stress tests explain why the gains are not confined to one metric. Salience weighting protects pathognomonic claims from dilution, *K* = 2 maximizes the retrieval trade-off, and graph masking produces a graded decline rather than an abrupt failure. Modality-specific preprocessing narrows the Messidor-2 external gap, while the inference profile shows that four-bit deployment fits within a 24-GB GPU. A deployment-ready prospective multicenter program can therefore be specified around calibration, subgroup safety, referral decisions, workflow time, and patient outcomes, with the same patient-clustered estimands used here.

## 6 Conclusion

We developed a comprehensive framework for detecting and reducing false information (hallucinations) when using large language models for ophthalmology called HalluciGuard-Eye. By combining salience-aware, graph-structured retrieval with visual-semantic alignment and hierarchical factuality scoring, we reduced hallucinations while preserving diagnostic performance and improved lesion-level reporting, negation, and severity accuracy. Public benchmark results are complemented by retrieval, patient-clustered, domain-shift, inference, and clinician-in-the-loop analyses conducted on real experimental data. Together, these results define a reproducible evaluation package for trustworthy AI-assisted ophthalmic diagnostics.

## Data Availability

The four public benchmark datasets analyzed in this study are available from their original sources: EyePACS at https://www.kaggle.com/c/diabetic-retinopathy-detection/data (subject to the Kaggle competition terms of use); Messidor-2 at https://www.adcis.net/en/third-party/messidor2 (for research and educational purposes, with acknowledgement of the LaTIM laboratory and the Messidor program partners); OCTDL at https://data.mendeley.com/datasets/sncdhf53xc/4 (CC BY 4.0; code at https://github.com/MikhailKulyabin/OCTDL); and the Kermany OCT dataset at https://data.mendeley.com/datasets/rscbjbr9sj/3 (CC BY 4.0). The retrospective single-center diabetic retinopathy cohort data are not publicly available because they contain patient-level protected health information restricted by institutional regulations; these data may be obtained from the corresponding author upon reasonable request, subject to ethical approval.
